# Having It out

**Published:** 1918-02-16

**Authors:** 


### The Hospital

The Workers' Newspaper of Administrative Medicine and Institutional Life, Administration, National Insurance and Health.

No. 1654, Vol. LXIII. SATURDAY, FEBRUARY 16, 1918. PRICE ONE PENNY

### HAVING IT OUT.

Give sorrow words: the grief that does not speak,
Whispers the o'er-fraught heart, and bids it break.

A couple of months ago Dr. Rivers read before the Royal Society of Medicine an interesting paper, the gist of which is expressed in the exhortation given by Malcolm to Macduff, and quoted above. The observation that it is often beneficial to give utterance to our woes is not altogether novel, therefore, but, like other ancient truths, it is apt to be forgotten, and to be overlaid with new teaching, and then it does us good to be reminded of them. Dr. Rivers states his case temperately and reasonably, without any of the fanaticism or optimism of the recent convert, or the discoverer of what is believed to be new, though, as in this case, it may be as old as the hills; and he does not fail to secure the sympathy and assent of his readers.

He uses the word "repression" to signify "the process whereby a person attempts to thrust out of his memory some part of his mental content." In another place he speaks of it as "the actual or voluntary process by which it is attempted to remove some part of the mental content out of the field of attention, with the aim of making it inaccessible to memory and producing the state of suppression." Using the word in this sense, repression, says Dr. Rivers, is not in itself a pathological process, nor is it necessarily the cause of pathological states. On the contrary, it is a necessary element in education and in all social progress. We heartily agree, and are pleased to record Dr. Rivers' opinion. He speaks of the pathological state that sometimes results from repression as akin to "dissociation." We are pleased to remark that in his own dissociation from certain Continental teaching there is nothing pathological, but much that is sane and healthy.

It is natural, he says, to thrust aside painful memories just as it is natural to avoid dangerous or horrible scenes in actuality, so that even if left to themselves most people would naturally strive to forget distressing memories or thoughts; but sufferers from shell-shock, of whom he was giving his experience are very far from being left to themselves. They are perpetually urged, not only by their relatives and friends, but also by their medical advisers, to banish all thoughts of war from their minds. In some cases, all conversation between patients or with visitors about the war is forbidden, and no doubt, within limits and in reason, the advice to avoid constant dwelling upon these topics is sound and good. Nothing, says Dr. Rivers, annoys a nervous patient more than the continual inquiries of his relatives and friends about his experiences at the Front. The inquiries are injudicious, not only because they awaken painful memories, but also because of the mental strain they put upon the patient to describe the indescribable and explain the inexplicable. When veteran and competent newspaper reporters, who have had the training of a lifetime in the art of description, acknowledge their inability to convey any adequate notion of the experiences of modern trench warfare to their readers, it is manifestly absurd to expect an unlettered Tommy, or even a well-educated officer with no special training in this kind of work, to do so; and the effort to accomplish the impossible is painful and exhausting. When it is constantly or even frequently repeated, it cannot fail to be pernicious.

But though the constant dwelling upon the war experiences that have brought about the morbid condition cannot fail to be pernicious, and are found in practice to be very pernicious, it does not follow that the opposite course of driving these experiences out of the mind, or trying to do so, is wholly beneficial or the right course to take. It is the course that is usually advised, and although the advice strikes us as rather like advising a man with the convulsed hand of writer's cramp or the *tic convulsif* of the sterno-mastoid to keep the convulsed part still, yet to a certain extent the advice can be followed. The painful memory can be driven from the direct line of mental sight into the remoter margin of the field of consciousness; but beyond this it cannot be driven. It is always there, and it is always tending to return to the region of the yellow spot, from which it can be kept at a distance only by constant strain and effort. This strain and effort, which Dr. Rivers calls repression, is the harmful process; and though the painful memories can, by means of strain and effort, be kept at the outer margin of consciousness as long as the effort can be sustained, yet the effort cannot always be sustained. As long as the mind is under the control of the will, as long as the attention can be kept concentrated upon other things, as long as the patient can engage in occupations, amusements, discussions, conversations, that occupy his attention and fill his thoughts, the objectionable memories can be kept out of the line of sight, and the patient appears, and is, cheerful and well. "But ah! there comes another day," the day, or rather the night, when the effort can no longer be sustained. During sleep the mind is no longer under the control of the will. During sleep the strain relaxes; and when sleep relaxes the strain the evil memories come surging back into the direct line of mental vision and occupy the whole field of consciousness. The more complete and the more successful the waking effort to keep them at a distance, the more complete, it seems, is their dominance when the control of the will is abolished and waking consciousness is superseded by dreaming consciousness. The dreams are terrible. The patient is afraid to go to sleep. He is terrified as the hour of sleep approaches. He is afraid to be left in the dark. Even before he goes to sleep the terrifying memories that he has kept at bay by action and occupation come crowding back and race through his mind in a pandemonium of dread; and when at length he sleeps, his dreams are frightful, and he wakes quaking, sweating, and it may be sobbing in his misery.

In such conditions Dr. Rivers has found that relief may be given by encouraging the patient to bring his trouble forward, look it straight in the face, talk it over, and in short "have it out." His doctor is to listen to him, to discuss the matter with him, to find, if he can, any element in the experience that may be comforting. In one case the awful memory that the patient succeeded in beating off in the daytime came back at night and infested his dreams, but Dr. Rivers succeeded. He suggested that the mangled state of the body was conclusive evidence of his friend having been killed instantaneously, and that this was better than dying after a long, lingering, painful, and repulsive illness, such as is too often the lot of those who receive mortal wounds. The patient accepted this view with alacrity, and thereafter, though he still sometimes dreamt of his friend, the dreams were no longer terrifying—a sane, sensible, and encouraging suggestion for treatment.

